# Corticosteroid therapy in a patient with cerebral amyloid angiopathy-related inflammation

**DOI:** 10.1186/1742-2094-10-39

**Published:** 2013-03-16

**Authors:** Akio Kimura, Takeo Sakurai, Nobuaki Yoshikura, Yuichi Hayashi, Masao Takemura, Hitoshi Takahashi, Takashi Inuzuka

**Affiliations:** 1 Departments of Neurology and Geriatrics, Gifu University Graduate School of Medicine, 1-1 Yanagido, Gifu, 501-1194, Japan; 2 Informative Clinical Medicine, Gifu University Graduate School of Medicine, 1-1 Yanagido, Gifu, 501-1194, Japan; 3 Department of Pathology, Brain Research Institute, Niigata University, 1-757 Asahimachi-dori, Chuo-ku, Niigata City, Japan

**Keywords:** Amyloid angiopathy, Antibody, Brain, Encephalopathy, Immunology

## Abstract

We studied longitudinal changes of the levels of anti-amyloid β (anti-Aβ) antibody, amyloid β (Aβ) protein, and interleukin 8 (IL-8) in cerebrospinal fluid (CSF) of a patient with cerebral amyloid angiopathy-related inflammation (CAA-ri) in whom steroid treatment resulted in clinical improvement. The diagnosis of CAA-ri was established with brain biopsy. Levels of anti-Aβ 42 antibody, Aβ 40, Aβ 42 and IL-8 in CSF were measured in the CAA-ri patient at 23 time points in the 8-month clinical course. These CSF samples were divided into 2 groups: those obtained before (n = 12) and those after (n = 11) oral corticosteroid therapy was started. We compared these levels between CSF samples obtained before and after therapy. The mean levels of anti-Aβ 42 antibody and IL-8 were significantly higher in CSF samples of the CAA-ri patient before oral corticosteroid therapy than those after therapy. A positive correlation was noted between levels of anti-Aβ 42 antibodies and IL-8 in CSF of this patient. There were no significant differences of mean levels of Aβ 40 and Aβ 42 between CSF samples obtained before and after oral corticosteroid therapy. It was possible that the autoinflammatory process with anti-Aβ 42 antibodies and IL-8 may have been involved in the pathogenesis of CAA-ri, and that corticosteroid therapy directly affected levels of anti-Aβ 42 antibody and IL-8. In summary, CAA-ri encephalopathy is a relapsing or progressive disorder and may be treatable by adequate immunosuppressive therapy. The anti-Aβ 42 antibody in CSF is a useful biological marker for therapeutic monitoring of CAA-ri.

## Background

Cerebral amyloid angiopathy-related inflammation (CAA-ri) is characterized by sub-acute confusion, progressive cognitive decline, seizure or headaches; reversible focal subcortical and/or cortical T2 hyperintensities on magnetic resonance imaging (MRI); and neuropathological evidence of cerebral amyloid angiopathy (CAA) and associated vascular or perivascular inflammation [1-3]. Although the apolipoprotein E ε4/ε4 genotype is strongly associated with CAA-ri [1], the pathogenesis of CAA-ri is unknown. Clinical reports have noted a response to immunosuppressive treatment, suggesting that this syndrome may be a treatable form of CAA [4,5]. The syndrome may be diagnosed non-invasively, based on a characteristic combination of clinical and radiographic features [6], but the only way to confirm the diagnosis is through brain biopsy. Two previous studies have demonstrated the presence of anti-amyloid β (anti-Aβ) antibodies associated with CAA-ri [7,8]. In the present study, we longitudinally analyzed anti-Aβ antibody, amyloid β (Aβ) protein and interleukin 8 (IL-8) in cerebrospinal fluid (CSF) samples of a patient who had the diagnosis of CAA-ri confirmed with brain biopsy samples.

## Case presentation and methods

**Figure 1:**
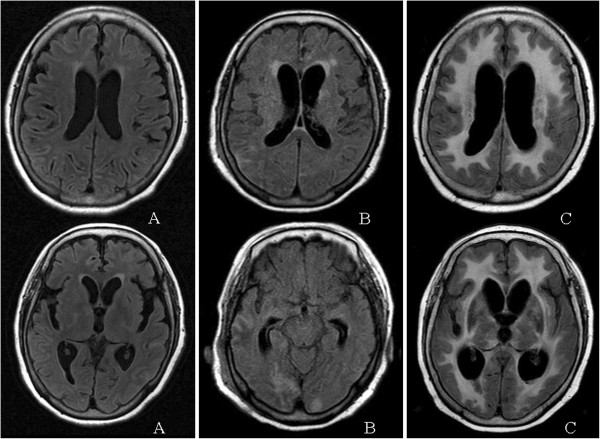
**Axial fluid-attenuated inversion recovery (FLAIR) brain MRI scans of the patient with CAA-ri.** (**A**) At time of admission, there was increased signal intensity in the leptomeninges and sulci; (**B**) At 49 days after admission, there were more extensive hyperintensity lesions in the leptomeninges and sulci, and there was asymmetric subcortical white matter of the occipital lobes; (**C**) At 195 days after admission, the hyperintensity lesions extended diffusely through the white matter.

A 72-year-old woman with an unremarkable past medical history presented with a 5-month history of headache and appetite loss. She developed mild fever and was admitted to a local hospital. After admission, she developed a disturbance of consciousness and was transferred to our hospital. Neurological examination showed somnolence, meningeal signs and rigidity in both elbow joints. Laboratory tests showed increased white blood cell count (17.7 × 10^9^/L; normal range: [3.3 to 7.9] × 10^9^/L) and neutrophil cell count (16.0 × 10^9^/L; normal range; [1.5 to 5.9] × 10^9^/L). The serum C-reactive protein level was elevated (12.5 mg/dL; normal: <0.20 mg/dL). Serum antibodies were absent, including antinuclear, anti-SS-A, anti-SS-B, anti-DNA, anti-Sm, anti-RNP, and perinuclear and cytoplasmic anti-neutrophil cytoplasmic antibodies. The CSF had 243 cells/mm^3^ (mononuclear cells: 98 cells/mm^3^; polynuclear cells: 145 cells/mm^3^) and elevated total protein concentration (133 mg/dL). The immunoglobulin G (IgG) index was elevated (0.89; normal: ≤0.73). No CSF oligoclonal IgG bands were detected. Cultures of CSF showed no bacteria, mycobacteria or fungi. Cryptococcus and aspergillus antigen tests were negative. Polymerase chain reaction (PCR) was negative for *Herpes simplex virus* and *Mycobacterium avium* complex. The nested PCR for detection of *Mycobacterium tuberculosis* was negative. Bacteria, mycobacteria and fungi were not detected from CSF or brain tissue using a broad-range PCR assay targeting the 16S ribosomal RNA gene regions of bacteria, heat shock protein (hsp65) gene regions of mycobacterium species, and the internal transcribed spacer (ITS) regions of fungi. Brain MRI scan (Signa Excite Xl Twin Speed 1.5 T system, GE Healthcare, Milwaukee, WI, USA) showed increased signal intensity on fluid-attenuated inversion recovery (FLAIR) images in the leptomeninges and sulci (Figure 1).

On admission to our hospital the patient’s consciousness deteriorated further and she required mechanical respiratory treatment. She was treated with intravenous antibiotics, antifungal drugs and acyclovir with intravenous methylprednisolone (1,000 mg/day for 3 days without subsequent oral corticosteroids). Her symptoms transiently improved but her consciousness progressively deteriorated again, and she required further mechanical respiratory treatment. At 49 days after admission, a repeat MRI scan with T2-weighted and FLAIR images showed more extensive hyperintensity lesions in the leptomeninges and sulci, asymmetric subcortical white matter of the occipital lobes, and hyperintensity lesions that extended through the white matter (Figure 1).

**Figure 2:**
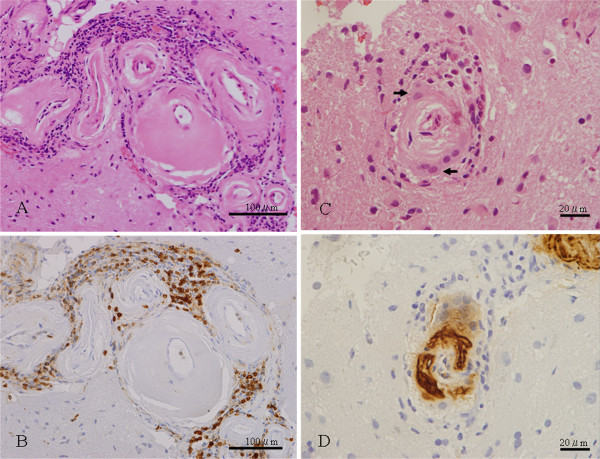
**Neuropathological findings of the biopsy specimen from the right parietal lobe of the patient with CAA-ri.** (**A**) Multifocal lymphocytic infiltrates around the leptomeningeal-parenchymal blood vessels, with hyaline thickening and splitting of the walls (double-barrel formation) (hematoxylin-eosin); (**B**) The perivascular lymphocytic infiltrates consisting primarily of UCHL1 + T cells (serial section of [A]; UCHL1 immunostain); (**C**) An affected blood vessel, showing perivascular multinucleated giant cells (arrows) (hematoxylin-eosin); (**D**) Dense deposition of Aβ protein in the affected blood vessel wall (serial section of [C]; Aβ immunostain).

At 77 days after admission, stereotactic brain biopsy of the right parietal lobe was performed. Neuropathological examination revealed advanced CAA. Some of the affected blood vessels showed inflammatory granulomatous vasculitis with perivascular infiltration of lymphocytes, multinucleated giant cells, and reactive astrocytosis in the surrounding brain parenchyma (Figure 2). Mild, lymphocytic infiltration was noted in a piece of the dura mater sampled. Immunostaining revealed T cell-predominant perivascular infiltrates and Aβ deposits in the affected blood vessel walls (Figure 2). The diagnosis of CAA-ri was made and the patient was treated with intravenous methylprednisolone (1,000 mg/day for 3 days) followed by oral corticosteroids. The symptoms gradually improved and she no longer required mechanical ventilation. According to the reduction of oral corticosteroid, the total protein concentration in CSF was elevated again. Then we added the cyclophosphamide. At 246 days after admission, she was transferred to another hospital.

### Analysis of anti-amyloid β 42 antibody, amyloid β 40, amyloid β 42 and interleukin 8 levels in cerebrospinal fluid

We measured the levels of anti-Aβ 42 antibody, Aβ 40 and Aβ 42 in CSF at 23 time points in the patient with CAA-ri during the 8-month clinical course by Enzyme-Linked Immunosorbent Assay (ELISA). These CSF samples were divided into 2 groups: those obtained before (0 to 103 days after admission; n = 12) and those after (≥116 days after admission; n = 11) oral corticosteroid therapy was started.

The levels of anti-Aβ 42 antibody, Aβ 40, and Aβ 42 were measured in CSF samples of control subjects. These subjects were 14 age-matched patients with Alzheimer’s disease and 7 patients with multiple sclerosis who were randomly selected patients from the Department of Neurology and Geriatrics, Gifu University Graduate School of Medicine, Japan (Table 1). All patients with multiple sclerosis met diagnostic criteria for the 2010 revision to the McDonald criteria [9]. These patients had relapsing and remitting type, and CSF was sampled during the relapse phase. At the time of CSF sampling, 2 patients with multiple sclerosis were treated with interferon β1b, 1 patient was treated with oral prednisolone (5 mg/d), and 1 patient was treated with mizoribine (50 mg/day). All patients with Alzheimer’s disease met Diagnostic and Statistical Manual of Mental Disorders (DSM)-IV criteria [10]. No patients with Alzheimer’s disease were treated with immunosuppressants that could have influenced the level of anti-Aβ 42 antibodies and IL-8.

**Table 1:** Demographic and clinical features of control subjects*

**Feature**	**Alzheimer’s disease**	**Multiple sclerosis**
No. patients	14	7
Gender, female / male	7 / 7	5 / 2
Age at evaluation (y)	73 ± 9	42 ± 9
Age at onset (y)	72 ± 9	30 ± 10
Disease duration (y)	1.9 ± 0.7	12 ± 8
Immunomodulatory therapy^†^	None	Prednisolone, 5 mg/day (1 patient); interferon β1b, 8 MIU/every other day (2 patients); mizoribine, 50 mg/day (1 patient)

*Data reported as number or mean ± SD.

^†^At time of sampling of cerebrospinal fluid.

We measured the levels of anti-Aβ 42 antibody, Aβ 40 and Aβ 42 in 2 serum samples obtained before oral corticosteroid therapy in the patient with CAA-ri by ELISA. We also measured these levels in serum samples of 7 patients with multiple sclerosis and 10 of 14 patients with Alzheimer’s disease. The CSF and serum samples of all subjects were collected and stored at −30°C. Commercially available kits were used for measuring levels of anti-Aβ 42 antibody (Human anti-Aβ42 ELISA kit, DRG International, Mountainside, NJ, USA), Aβ 40 and Aβ 42 (Human Aβ 40 and Aβ 42 brain ELISA kits, Millipore, Billerica, MA, USA). The patient with CAA-ri had polynuclear pleocytosis. Therefore, we also measured the levels of the neutrophil chemotactic factor IL-8 in CSF, which is a major mediator of the inflammatory response, in the CAA-ri patient and control subjects. The IL-8 levels were measured with a commercially available kit (Human IL-8 ELISA kit, eBioscience, San Diego, CA, USA). These commercial kits used solid phase ELISA that was based on the sandwich principle. The level of anti-Aβ 42 antibody was reported as units, which were defined commercially from a calibration curve as an indicator of concentration (not as units in enzymatic assays that describe turnover rate of substrate under defined conditions and time). The levels of Aβ40, Aβ42 and IL-8 were reported as concentrations (pg/mL). This study was approved by the institutional review board of Gifu University Graduate School of Medicine, Gifu City, Japan.

### Data analysis

The Mann–Whitney test was used to compare the mean levels of anti-Aβ 42 antibody, Aβ 40, Aβ 42 and IL-8 between CSF samples obtained before and after oral corticosteroid therapy was started. The Spearman rank correlation was used to assess the correlation between the anti-Aβ 42 antibody level, IL-8 level, total cell count and total protein concentration in the CSF samples of the patient with CAA-ri. Statistical significance was defined by *P* ≤0.05.

## Results

### Comparison of the levels of anti-amyloid β 42 antibody, amyloid β 40, amyloid β 42 and interleukin 8 in cerebrospinal fluid samples obtained before and after oral corticosteroid therapy in the patient with cerebral amyloid angiopathy-related inflammation

**Figure 3:**
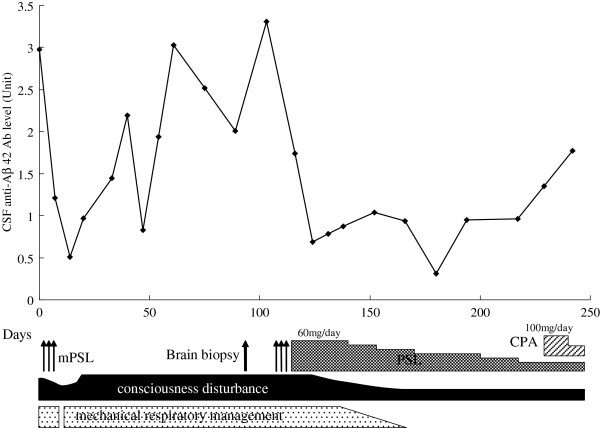
**Clinical course and longitudinal changes in levels of anti-Aβ 42 antibody in CSF of the patient with CAA-ri.** CPA, cyclophosphamide; Days: time from admission; mPSL, methylprednisolone; PSL, prednisolone.

**Figure 4:**
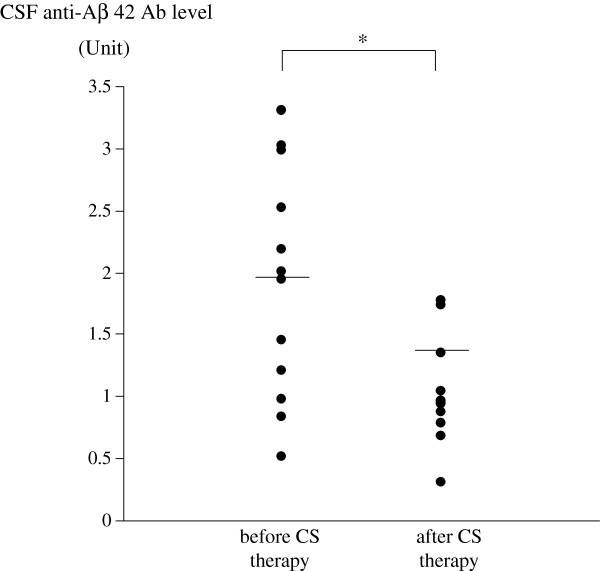
**Levels of anti-Aβ 42 antibody in CSF of the patient with CAA-ri before and after oral corticosteroid therapy.** Mean values are indicated by horizontal lines. *P <0.01.

**Figure 5:**
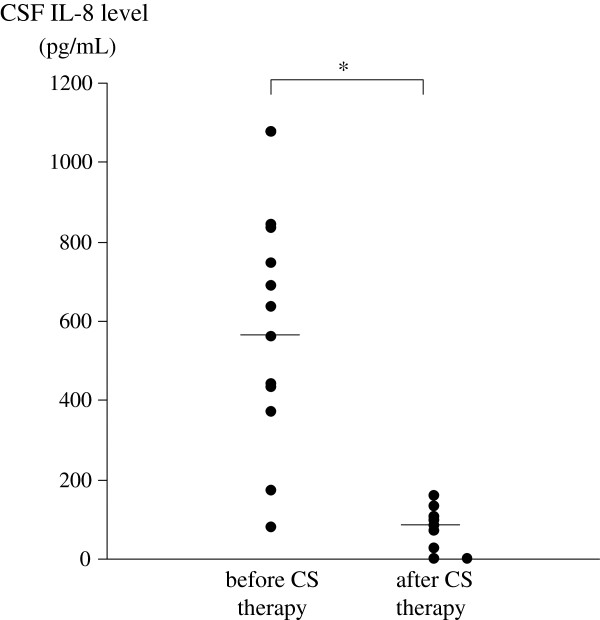
**Levels of IL-8 in CSF of the patient with CAA-ri before and after oral corticosteroid therapy.** Mean values are indicated by a horizontal line. *P <0.0005.

**Figure 6:**
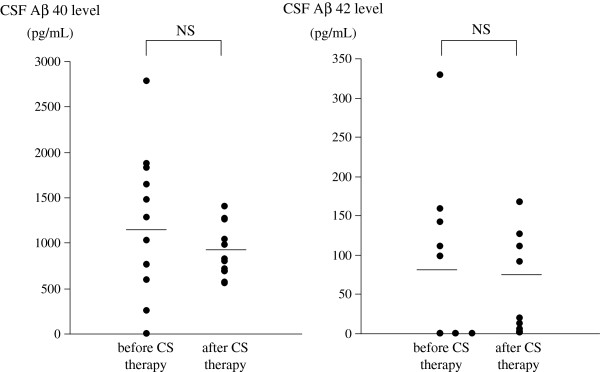
**Levels of Aβ 40 and Aβ 42 in CSF of the patient with CAA-ri before and after oral corticosteroid therapy.** Mean values are indicated by a horizontal line. NS, not significant (*P* >0.05).

In CSF, the levels of anti-Aβ 42 antibodies varied widely during the first 4 months of the clinical course (Figure 3). The mean level of CSF anti-Aβ 42 antibody in this patient was significantly greater before oral corticosteroid therapy than after (Figure 4). The mean level of IL-8 also was significantly greater in CSF samples obtained before oral corticosteroid therapy than those obtained after (Figure 5). There were no significant differences of mean levels of Aβ 40 and Aβ 42 between CSF samples obtained before and after oral corticosteroid therapy (Figure 6).

### Comparison of the levels of anti-amyloid β 42 antibody, amyloid β 40, amyloid β 42 and interleukin 8 in cerebrospinal fluid and serum samples between the patient with cerebral amyloid angiopathy-related inflammation and control subjects

We evaluated the levels of anti-Aβ 42 antibody, Aβ 40, Aβ 42 and IL-8 in CSF or serum samples of the CAA-ri patient and control subjects (Table 2). The mean anti-Aβ 42 antibody level of CSF samples obtained before oral corticosteroid therapy was greater in the CAA-ri patient than control subjects. We detected no increase in the anti-Aβ 42 antibody levels of serum samples obtained before oral corticosteroid therapy in the CAA-ri patient compared with those in control subjects.

**Table 2:** Levels of anti-Aβ 42 antibody, Aβ 40, Aβ 42 and IL-8 in CSF or serum of the patient with CAA-ri and control subjects*

**Disease**		**No. samples**	**Anti-Aβ 42 Ab level (U)**	**Aβ 40 (pg/mL)**	**Aβ 42 (pg/mL)**	**IL-8 (pg/mL)**
CAA-ri (1 patient)	All values CSF	23	1.5 ± 0.9	1030 ± 640	72 ± 8	340 ± 330
		2	38 ± 24	1490 ± 327	<0.005	-
	Before oral corticosteroids CSF	12	1.9 ± 0.9^†^	1130 ± 850	78 ± 101	570 ± 290^‡^
		2	38 ± 24	1490 ± 327	<0.005	-
	After oral corticosteroids CSF	11	1.4 ± 0.4^†^	920 ± 290	65 ± 69	80 ± 52^‡^
Alzheimer’s disease (14 patients) CSF		14	0.8 ± 0.8	4990 ± 2080	540 ± 160	12 ± 46
		10	29 ± 19	1330 ± 1040	52.7 ± 120	-
Multiple sclerosis (7 patients) CSF		7	1.2 ± 0.9	4180 ± 2080	770 ± 450	0 ± 0
		7	54 ± 22	683 ± 785	<0.005	-

*Data reported as mean ± SD. Ab: Antibody; Aβ: Amyloid β; CAA-ri: Cerebral amyloid angiopathy-related inflammation; CSF: Cerebrospinal fluid; IL-8: Interleukin 8.

^†^Anti-Aβ 42 Ab level in CSF: difference between levels before and after oral corticosteroids, *P* ≤0.01.

^‡^IL-8 level in CSF: difference between levels before and after oral corticosteroids, *P* ≤0.0005.

The mean Aβ 40 and Aβ 42 levels of the CSF samples obtained before and after oral corticosteroid therapy, and all values combined, were lower in the CAA-ri patient than control subjects. The mean IL-8 level of the CSF samples obtained before and after oral corticosteroid therapy, and all values combined in the CAA-ri patient, were greater than those in control subjects.

### Correlation between the anti-Aβ 42 antibody level, IL-8 level, total cell count and total protein concentration in the cerebrospinal fluid samples of the patient with cerebral amyloid angiopathy-related inflammation

**Figure 7:**
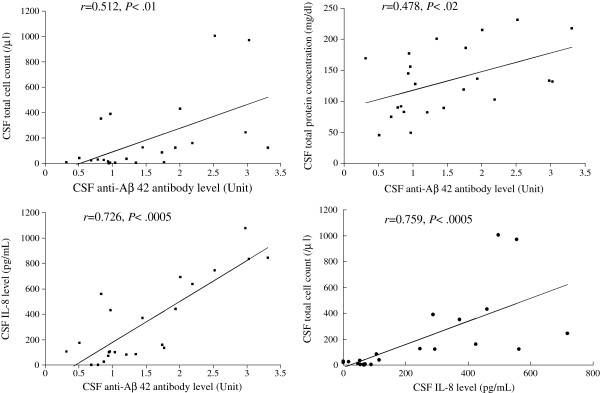
Correlation between the anti-Aβ 42 antibody level, IL-8 level, total cell count and total protein concentration in the CSF samples of the patient with CAA-ri.

We evaluated the correlation between the anti-Aβ 42 antibody level, IL-8 level, total cell count and total protein concentration in the CSF samples of the patient with CAA-ri (Figure 7). The CSF total cell count, total protein concentration and IL-8 level were significantly correlated with the CSF anti-Aβ 42 antibody level in the CAA-ri patient. The CSF total cell count also was significantly correlated with the CSF IL-8 level.

## Discussion

This study showed that the mean level of anti-Aβ 42 antibody in the patient with CAA-ri was significantly greater in CSF samples obtained before starting oral corticosteroid therapy than those obtained after. After oral corticosteroid therapy was started in the present CAA-ri patient, the level of anti-Aβ 42 antibody significantly decreased and the clinical symptoms improved. This supports the concept that anti-Aβ 42 antibodies in CSF may mediate the autoimmune reaction occurring in CAA-ri, and that corticosteroids had a therapeutic effect on elevated anti-Aβ 42 antibody in the CSF of the patient with CAA-ri.

The mean level of anti-Aβ 42 antibody of CSF samples obtained before oral corticosteroid therapy was greater in the CAA-ri patient than control subjects. Using CSF/serum quotient diagrams (Reibergrams), we evaluated whether this increased level of anti-Aβ 42 antibody of CSF samples obtained before oral corticosteroid therapy in the CAA-ri patient was a result of blood–brain barrier damage or specific intrathecal synthesis [11]. The increased level of anti-Aβ 42 antibody was not explained by blood–brain barrier damage, but was caused by specific intrathecal synthesis of anti-Aβ antibodies (quotient-anti-Aβ 42 antibody: 54.2 × 10^-3^; quotient-total IgG: 16.9 × 10^-3^). This confirms the findings of recent reports [7,8]. However, the present study is limited because there was only 1 patient with CAA-ri. Future studies including a larger number of CAA-ri patients and control subjects are expected.

The radiographic abnormalities of the present patient with CAA-ri progressed and did not improve despite corticosteroid treatment. The beneficial effects of corticosteroid treatment may vary with the pathological subtype of CAA-ri. There have been 2 subtypes of CAA-ri described, including a non-vasculitic form (perivascular infiltration) and a vasculitic form (transmural granulomatous angiitis) [4]. These pathological forms can occur together in the same patient [12]. Neuropathological findings of the present study suggested that our patient had concurrent perivascular infiltration and transmural granulomatous angiitis. In general, patients with transmural granulomatous angiitis may not benefit as much as patients with perivascular infiltration by immunosuppressive therapy. A previous long-term follow-up study showed that CAA-ri can be a relapsing or progressive disorder [6]. The present CAA-ri patient was treated twice with intravenous methylprednisolone pulse therapy. The CSF level of anti-Aβ 42 antibody was immediately and strongly reduced after intravenous methylprednisolone pulse therapy. However, this antibody level increased within a few weeks after the first intravenous methylprednisolone pulse therapy that was not followed with oral corticosteroids. The present study showed that this antibody level was progressively increasing, according to the reduction of oral corticosteroid. If the CSF level of anti-Aβ 42 antibody correlates with the ongoing autoimmune process, the patient’s history suggests that the inflammatory process resumed after the first methylprednisolone pulse, and it may be advisable to place some CAA-ri patients on immunosuppressive treatment. Repeat measurement of this antibody level in CSF may help monitor the course of the disease and efficacy of treatment.

The mean IL-8 level in the present CAA-ri patient was also significantly greater in CSF samples obtained before than those after oral corticosteroid therapy (Table 2), and were greater in the CAA-ri patient than in control subjects (Table 2). Moreover, a positive correlation was noted between levels of anti-Aβ 42 antibodies and IL-8 in CSF of the CAA-ri patient (Figure 7). Small heat shock proteins are associated with Aβ deposits in CAA and induce a cerebral inflammatory reaction [13]. These small heat shock proteins induce the production of IL-8 by human leptomeningeal smooth muscle cells and human brain astrocytes *in vitro*[13]. Therefore, an autoinflammatory process with anti-Aβ 42 antibodies and IL-8 may be involved in the pathogenesis of CAA-ri. However, caution is advised in the interpretation of these findings because the results of this study may have been influenced by an unusual presentation for this patient. The polynuclear pleocytosis in our patient was atypical compared with previous reports of CAA-ri with normal to mild mononuclear pleocytosis [1,3]. A possible explanation for this finding is that the present patient may have had more severe leptomeningeal vessel inflammation than previously reported CAA-ri patients. Another possibility is that the initial presentation was caused by complications of viral meningoencephalitis. Inflammatory markers including IL-8 may have been high if this patient had pre-existing viral meningoencephalitis. More studies are needed to clarify the pathological role of IL-8 in CAA-ri.

The mean levels of Aβ 40 and Aβ 42 in CSF were lower in the CAA-ri patient than control subjects (Table 2). This confirms the findings of a previous case report in which a patient with CAA-ri had a low level of Aβ 42 level in CSF [7] and other reports that showed low levels of Aβ 40 and Aβ 42 in CSF of patients with CAA [14,15]. Previous histological data support the deposition of Aβ 40 and Aβ 42 proteins in CAA [16]. In CAA, both Aβ 40 and Aβ 42 may be trapped in the cerebral vasculature, and deplete these peptides from CSF. The diagnosis of CAA-ri requires recognition of CAA [1,17]. Echo gradient imaging and presence of microbleeds could be used as a useful screening tool in CAA. We could not perform this imaging in the present study and could not make the diagnosis of CAA-ri until the brain biopsy was performed. Echo gradient imaging can help speed up the diagnostic process and prompt an earlier biopsy. It is possible that the low level of Aβ 40 and Aβ 42 in CSF could be an alternative screening marker of CAA and CAA-ri. Further studies are required to assay levels of Aβ 40 and Aβ 42 in CSF in more patients with CAA and CAA-ri.

## Conclusions

The encephalopathy CAA-ri is treatable by adequate immunosuppressive therapy. We demonstrated the possible therapeutic effect of corticosteroids on elevated anti-Aβ 42 antibody and IL-8 in CSF of the patient with CAA-ri. It may be helpful to place some CAA-ri patients on immunosuppressive therapy, and anti-Aβ 42 antibody in CSF may be a potentially useful biological marker for the therapeutic monitoring of CAA-ri.

## Consent

Written informed consent was obtained from the next of kin of the patient for publication of this case report and any accompanying images. A copy of the written consent is available for review by the Editor-in-Chief of this journal.

## Abbreviations

Aβ: Amyloid β; CAA: Cerebral amyloid angiopathy; CAA-ri: Cerebral amyloid angiopathy-related inflammation; CSF: Cerebrospinal fluid; ELISA: Enzyme-linked immunosorbent assay; FLAIR: Fluid-attenuated inversion recovery; IL: Interleukin; MRI: Magnetic resonance imaging

## Competing interests

The authors declare that they have no competing interests.

## Authors’ contributions

AK designed this article, analyzed the data and drafted the manuscript. TS, NY and YH collected the data. MT carried out the immunoassays. HT carried out the pathological examination. TI helped to analyze the data and draft the manuscript. All authors read and approved the final manuscript.

## Authors’ information

AK, TS, NY, YH and TI are members of the Departments of Neurology and Geriatrics, Gifu University Graduate School of Medicine, and MT is a member of the Informative Clinical Medicine, Gifu University Graduate School of Medicine. HT is a member of the Department of Pathology, Brain Research Institute, Niigata University.
